# A Novel Method for Muscle Elongation in Myalgia with Naqvi's-Dynamic Electrical Therapy Approach (DELTA)©: The First-Ever Case Report

**DOI:** 10.7759/cureus.23075

**Published:** 2022-03-11

**Authors:** Waqar M Naqvi, Maliha Fatima Quraishi, Sakshi P Arora, Chanan Goyal

**Affiliations:** 1 Directorate of Research, NKP Salve Institute of Medical Sciences and Research Center, Nagpur, IND; 2 Community Health Physiotherapy, Datta Meghe Institute of Medical Sciences, Ravi Nair Physiotherapy College, Wardha, IND; 3 Musculoskeletal Physiotherapy, MGM School of Physiotherapy, Aurangabad, IND; 4 Paediatric Physiotherapy, Government Physiotherapy College, Raipur, IND; 5 Paediatric Physiotherapy, Datta Meghe Institute of Medical Sciences, Ravi Nair Physiotherapy College, Wardha, IND

**Keywords:** neck pain, trapezius myalgia, naqvi’s-dynamic electrical therapy approach, electrotherapy, physiotherapy, interferential therapy

## Abstract

Lifestyle-related neck and shoulder pain can be attributed to trapezius myalgia (TM) in a significant number of cases. Apart from pain, manifestations of TM include tightness of the trapezius muscle, especially in the upper fibres. Naqvi's-Dynamic Electrical Therapy Approach (Naqvi's-DELTA)© is a novel electrotherapeutic intervention based on the principle of myofibril elongation obtained by interference of poled vector current that is moved along the length of muscle fibres. A 22-year-old male approached the physiotherapy outpatient department (OPD) with the chief complaint of persistent neck pain and stiffness for three years that adversely affected his activities of daily living (ADLs). The pain was evaluated using a visual analog scale (VAS), restrictions in the range of motion (ROM) were determined by the cervical range of motion (CROM) device, and limitations in ADLs were assessed by neck disability index (NDI). Naqvi's-DELTA© was administered once a day for seven days, with each session lasting for 15 minutes. After the intervention, an evident beneficial effect was noted in all outcomes measures suggesting that this novel method was effective in decreasing pain, stiffness, and limitations in ADLs. Further investigation to explore this method for myalgia management is warranted.

## Introduction

Trapezius myalgia (TM) characteristically signifies pain, stiffness, and tightness of the trapezius muscle with more often involvement of upper fibres. The lifestyle nowadays is resulting in chronic neck and shoulder pain engulfing 10-20% of the adult population [1]. TM marks its presence with pain, stiffness, taut bands of myofibrils, tender nodules along the course of muscle fibres and fascia, which are also referred to as myofascial trigger points and often result in a restricted range of motion (ROM) [2,3].

The Naqvi's-Dynamic Electrical Therapy Approach (Naqvi's-DELTA)© is a novel approach of interfering the vector current along the length of the muscle fibres in a dynamic fashion. The bifurcated electrode leads are used for quadripolar dynamic application of electrical interference. The selection of electrodes is based on the principle that the size of the electrode is inversely related to the current density; as the size of the electrode decreases, the current density increases. The probes are fixed diagonally and are symmetrically separated by an insulator with a high dielectric constant to prevent the concentration of the current. The Aquasonic gel is used to reduce the skin impedance, which is inversely proportional to the stimulation frequency, and the probe unit is moved along the affected muscle fibres following circular, transverse, or figure of eight graphic patterns. The modulation of the current is formulated using the interference of two medium-frequency (1kHz-150kHz) currents producing a beat frequency deep in the tissues that results in the low-frequency effect [4]. The four-pole-vector mode is used for current administration with the triangular sweep pattern along with the beat frequency for 15 minutes. The intensity of the current delivered is according to the tolerance of the patient. The therapist dynamically moves the probe unit along the length of the muscle fibres with the vector interference within the four poles of the probe unit module.

The Naqvi's-DELTA© incorporates the depolarization of tissue without concentrating the current at one specific point of the muscle. Being novelty, the efficacy of the Naqvi's-DELTA© was investigated on a young adult with neck and shoulder pain. The treatment protocol was synthesized on the basis of the normal physiological response of the tissue to depolarisation during the static electrical current approach. The primary outcome measures used were the visual analog scale (VAS) for pain intensity [5], the cervical range of motion (CROM) device for the restricted ROM [6], and the secondary outcome measure used was the neck disability index (NDI) for limitation in activities of daily living (ADLs) [7].

## Case presentation

A 22-year-old male visited the physiotherapy outpatient department (OPD) with a chief complaint of pain and stiffness in the neck and shoulder region for three years, which gradually worsened and affected his ADLs and consequently restricted his participation.

Clinical findings 

The onset of pain was gradual, with the site being the posterior aspect of the neck without radiation. The character of pain was continuous with a deep dull aching type, and the intensity on VAS was 5.1 at rest and 7.4 during movements of the shoulder and neck. The aggravating factors included any neck movement, and the relieving factors were rest and analgesics. 

The patient was demonstrating severe stiffness leading to the restricted cervical ROM. The ROMs were assessed (Table 1) using CROM device consecutively for seven days before and after the treatment. The intensity of pain and the NDI score for limitations in ADLs were also assessed before and after the treatment (Table 2).

**Table 1:** Active ROM before and after the treatment consecutively for seven days using CROM device R - right; L - left; ROM - range of motion; CROM - cervical range of motion

Neck ROM	Flexion		Extension		Rotation		Lateral flexion	
	Pre	Post	Pre	Post	Pre	Post	Pre	Post
Day 1	0-20º	0-40º	0-15º	0-45º	R: 0-30º	R: 0-42º	R: 0-15º	R: 0-30º
					L: 0-35º	L: 0-39º	L: 0-21º	L: 0-35º
Day 2	0-44º	0-55º	0-35º	0-60º	R: 0-42º	R: 0-55º	R: 0-35º	R: 0-43º
					L: 0-38º	L: 0-50º	L: 0-35º	L: 0-44º
Day 3	0-50º	0-60º	0-40º	0-60 º	R: 0-50º	R: 0-60º	R: 0-40º	R: 0-44º
					L: 0-54º	L: 0-58º	L: 0-39º	L: 0-45º
Day 4	0-50º	0-60º	0-55º	0-70º	R: 0-51º	R: 0-55º	R: 0-40º	R: 0-45º
					L: 0-48º	L: 0-60º	L: 0-45º	L: 0-45º
Day 5	0-54º	0-66º	0-60º	0-73º	R: 0-54º	R: 0-60º	R: 0-35º	R: 0-40º
					L:0-52º	L: 0-65º	L: 0-45º	L: 0-45º
Day 6	0-61º	0-70º	0-60º	0-69º	R: 0-69º	R: 0-72º	R: 0-45º	R: 0-47º
					L: 0-62º	L: 0-78º	L: 0-45º	L: 0-48º
Day 7	0-78º	0-85º	0-70º	0-77º	R: 0-88º	R: 0-92º	R: 0-46º	R: 0-50º
					L: 0-85º	L: 0-89º	L: 0-45º	L: 0-49º

**Table 2:** The improvement observed in the pain intensity and ADLs limitation VAS - visual analog scale; NDI - neck disability index; ADLs - activities of daily living

Outcome measure	Pre-treatment (day 1)	Post-treatment (day 7)
VAS	At rest: 5.1	At rest: 1.2
	During neck movement: 7.4	During neck movement: 1.8
NDI	52% (severe disability)	8% (no disability)

Timeline

On 7th July 2021, the patient visited physiotherapy OPD and underwent a baseline assessment and diagnosis. On the same day, the treatment was initiated with Naqvi's-DELTA©. The follow-up assessment was done and reported on 13th July 2021.

Diagnostic assessment

The condition of the patient presented the differential diagnoses of cervical radiculopathy, cervical spondylosis, or myalgia of the neck and shoulder. The pain was not radiating in nature, ruling out the possibilities of cervical radiculopathy. The Spurling's test was negative, which clinically ruled out cervical spondylosis. The patient was having pain, stiffness, and taut bands along the fibres of the muscle pointing towards myalgia of the neck and shoulder. While assessing the bands, the upper fibres of bilateral trapezius muscle were found to be primarily involved indicating towards trapezius myalgia.

Physiotherapeutic intervention

The Naqvi's-DELTA© was used with the parameters set for four-pole-vector with the treatment duration of 15 minutes. Channels one and two were set to deliver the current according to the patient's tolerance in continuous mode with a beat frequency ranging between 0-150 Hz with a triangular sweep pattern. The unit was moved in an overlapping circular manner dynamically along the course of the muscle. The Naqvi's-DELTA© covered the trigger points of the entire muscle along its course. The treatment protocol was continued for seven days, and each day the pre-treatment and post-treatment cervical ROM of the patient were noted.

Follow-up and outcomes

The administration of Naqvi's-DELTA© was undertaken for seven consecutive days, and the improvements noticed were analyzed for physiological reasons. The decrease in pain intensity and the increase in cervical ROM, accompanied with ease in ADLs in the patient with TM, were noted with the outcome measures (Tables 1-2). The patient was advised ergonomically to prevent the recurrence of the condition [8].

## Discussion

The Naqvi's-DELTA© has physiological effects involving the physical, chemical, and mechanical responses of the tissue to depolarisation at different levels. The sub-sensory level activates before reaching the threshold of an individual to tolerate the intensity of the current wherein the leukocytes and macrophages activate themselves and proliferation of epithelial cells occurs, accelerating the tissue healing process in myalgia [9]. After reaching the threshold of current, the sensory level activation depolarises Ab nerves with a phase duration of 1-100 µsec leading to substantia gelatinosa activation and, in turn, closes the pain gate relieving the pain [10]. The motor level stimulation depolarises the type-II motor neurons with a phase duration of 200-400 µsec, developing the tension and elongation in the muscle fibres releasing the enkephalin and endorphin, which in turn closes the pain gate [11]. The noxious level stimulation depolarises the Ad nerves with a phase duration of 1-100 msec which stimulates the central biasing mechanism closing the pain gate and pain perception. The Naqvi's-DELTA© recruits the large-diameter, fast-twitch muscle fibres leading to asynchronous contraction and elongation of the muscle fibres based on the number of pulses per second [12]. During the electrically induced muscle contraction, the Golgi tendon organ cannot override the developing tension within the musculotendinous unit followed by a faster onset of fatigue which is subsided in Naqvi's-DELTA© by moving the probe unit along the muscle fibres [13].

The physiological effects being compared with the static interferential therapy suggested that the dynamic movement of the probe unit module facilitated the muscle fibres contractibility, improved the blood supply without concentrating the current at a point, and facilitated the drainage of lactic acid accumulated as a metabolite in the myofibrils [14]. The electrical muscle elongation using the interference of the current has been reported as an effective treatment method for different musculoskeletal conditions [15].

## Conclusions

The first-ever case report has demonstrated the efficacy of Naqvi's-DELTA© on pain, tenderness, stiffness, restricted ROM, and limitation in ADLs for TM. The dynamic movement of the probe unit module with the vector-current has applied the physiological responses of muscle fibres to depolarisation. The improvement demonstrated has opened the opportunity for exploring the effectiveness of different electro-medical currents using Naqvi's-DELTA©.
